# Hexaaqua­zinc(II) dichloride bis­(hexa­methyl­enetetramine) tetra­hydrate

**DOI:** 10.1107/S1600536808024793

**Published:** 2008-08-06

**Authors:** Xin Jian Yao, Ya Wen Xuan, Wen Wu

**Affiliations:** a Department of Chemistry, Zhokou Normal University, Henan 466001, People’s Republic of China

## Abstract

The title compound, [Zn(H_2_O)_6_]Cl_2_·2C_6_H_12_N_4_·4H_2_O, has been prepared under mild hydro­thermal conditions. The Zn^II^ atom, located on a centre of symmetry, is coordinated by six water mol­ecules in a distorted octa­hedral coordination geometry. The hexa­methyl­enetetra­mine mol­ecule is not coordinated to Zn^II^ but links the Zn complexes *via* three O—H⋯N hydrogen bonds. The remaining N atom of the hexa­methyl­enetetra­mine mol­ecule is hydrogen-bonded to a solvent water mol­ecule. In the crystal structure, inter­molecular O—H⋯O, O—H⋯N and O—H⋯Cl hydrogen bonds link the components into a three-dimensional network.

## Related literature

For related compounds, see: Zhang *et al.* (2000 ▶).
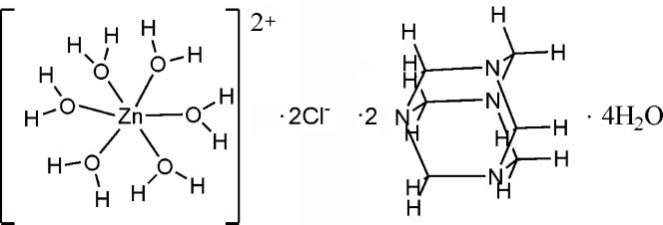


## Experimental

### 

#### Crystal data

[Zn(H_2_O)_6_]Cl_2_·2C_6_H_12_N_4_·4H_2_O
*M* *_r_* = 596.84
Triclinic, 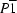

*a* = 9.345 (3) Å
*b* = 9.4176 (15) Å
*c* = 9.4535 (15) Å
α = 119.521 (1)°
β = 94.218 (2)°
γ = 100.969 (2)°
*V* = 697.0 (3) Å^3^
*Z* = 1
Mo *K*α radiation
μ = 1.13 mm^−1^
*T* = 291 (2) K
0.36 × 0.29 × 0.15 mm

#### Data collection

Bruker SMART CCD area-detector diffractometer
Absorption correction: multi-scan (*SADABS*; Sheldrick, 1996 ▶) *T*_min_ = 0.690, *T*_max_ = 0.849
5184 measured reflections
2576 independent reflections
2466 reflections with *I* > 2σ(*I*)
*R*_int_ = 0.018

#### Refinement

*R*[*F* ^2^ > 2σ(*F* ^2^)] = 0.038
*wR*(*F* ^2^) = 0.113
*S* = 1.05
2576 reflections
151 parameters
H-atom parameters constrained
Δρ_max_ = 0.51 e Å^−3^
Δρ_min_ = −0.68 e Å^−3^

### 

Data collection: *SMART* (Bruker, 2002 ▶); cell refinement: *SAINT* (Bruker, 2002 ▶); data reduction: *SAINT*; program(s) used to solve structure: *SHELXS97* (Sheldrick, 2008 ▶); program(s) used to refine structure: *SHELXL97* (Sheldrick, 2008 ▶); molecular graphics: *SHELXTL* (Sheldrick, 2008 ▶); software used to prepare material for publication: *SHELXTL*.

## Supplementary Material

Crystal structure: contains datablocks I, global. DOI: 10.1107/S1600536808024793/kp2186sup1.cif

Structure factors: contains datablocks I. DOI: 10.1107/S1600536808024793/kp2186Isup2.hkl

Additional supplementary materials:  crystallographic information; 3D view; checkCIF report

## Figures and Tables

**Table 1:** Hydrogen-bond geometry (Å, °)

*D*—H⋯*A*	*D*—H	H⋯*A*	*D*⋯*A*	*D*—H⋯*A*
O1—H1*W*⋯N3	0.82	2.05	2.827 (3)	158
O1—H2*W*⋯O5^i^	0.83	1.94	2.743 (3)	162
O2—H3*W*⋯N2^ii^	0.83	1.99	2.804 (3)	167
O2—H4*W*⋯O4^ii^	0.84	1.90	2.711 (3)	165
O3—H5*W*⋯Cl1	0.82	2.55	3.197 (2)	137
O3—H6*W*⋯N1^iii^	0.83	2.01	2.813 (3)	165
O4—H7*W*⋯Cl1	0.83	2.36	3.175 (2)	168
O4—H8*W*⋯N4^iv^	0.84	2.00	2.835 (3)	174
O5—H9*W*⋯Cl1	0.83	2.43	3.255 (3)	168
O5—H10*W*⋯Cl1^v^	0.83	2.38	3.213 (3)	175

Symmetry codes: (i) 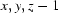; (ii) 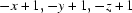; (iii) 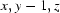; (iv) 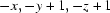; (v) 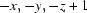.

**Fig. 1.**
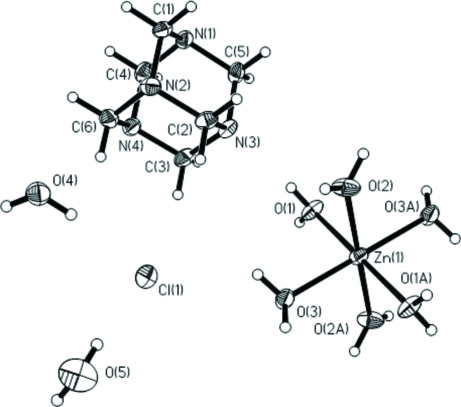
Plot of an asymmetric unit with the 30% probability ellipsoids.

**Fig. 2.**
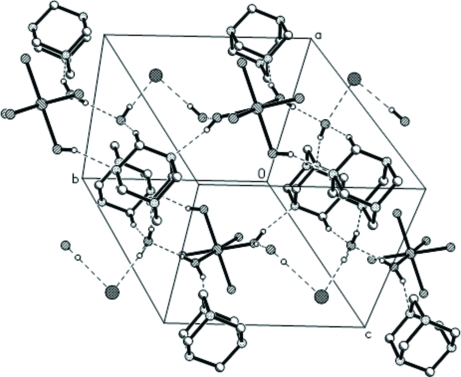
Three-dimensional network of hydrogen-bonding pattern with the motif R44(16) linking the cationic moieties with hexamine which are in turn interwoven with anionic moieties via water molecules.

## supplementary crystallographic information

### Comment

The asymmetric unit (Fig.1)  consists of one half of hexaaua Zn^II^ octahedron, one chloride ion, one uncoordinated neutral hexamethylenetetramine and two molecules of water of crystallization. The hexamethylenetetramine molecule is linked to the [Zn(H_2_O)_6_]^2+^*via* three O—H···N hydrogen bonds, while atom N3 of hexamethylenetetramine is hydrogen-bonded to O5 of the solvent water molecule. The Cl^-^ anions link to the [Zn(H_2_O)_6_]^2+^ and water of crystallization *via* O—H···Cl hydrogen bonding, Hydrogen bonding of these anionic and cationic frameworks results in the formation of a three-dimensional network (Table 1, Fig. 2).

### Experimental

All the reagents were of AR grade and used without further purification. C_6_H_12_N_4_ (1.401 g, 10 mmol) were dissolved in 50 mL H_2_O solution, then the resultant solution was added in 10 mL double-distilled water containing ZnCl_2_ (0.273 g, 2 mmol). The resulting solution was heated at 423 K for 96 h. After cooling to room temperature, block crystals were obtained in a yield up to 21.1%.

### Refinement

H atoms bonded to O atoms were located in a difference map and included in their 'as found' positions with *U*_iso_(H) = 1.5*U*_eq_(O). Other H atoms were positioned geometrically with C—H = 0.97 Å and with *U*_iso_(H)=1.2*U*_eq_(C). All H atoms were treated as riding.

### Crystal data

**Table:**

[Zn(H_2_O)_6_]Cl_2_·2C_6_H_12_N_4_·4H_2_O	*Z* = 1
*M**_r_* = 596.84	*F*_000_ = 316
Triclinic, *P*1	*D*_x_ = 1.422 Mg m^−^^3^
Hall symbol: -P 1	Mo *K*α radiation λ = 0.71073 Å
*a* = 9.345 (3) Å	Cell parameters from 2143 reflections
*b* = 9.4176 (15) Å	θ = 2.5–25.5º
*c* = 9.4535 (15) Å	µ = 1.13 mm^−^^1^
α = 119.5210 (10)º	*T* = 291 (2) K
β = 94.218 (2)º	Block, colorless
γ = 100.969 (2)º	0.36 × 0.29 × 0.15 mm
*V* = 697.0 (3) Å^3^	

### Data collection

**Table:**

Bruker SMART CCD area-detector diffractometer	2576 independent reflections
Radiation source: fine-focus sealed tube	2466 reflections with *I* > 2σ(*I*)
Monochromator: graphite	*R*_int_ = 0.018
Detector resolution: 0 pixels mm^-1^	θ_max_ = 25.5º
*T* = 291(2) K	θ_min_ = 2.5º
φ and ω scans	*h* = −11→11
Absorption correction: multi-scan(SADABS; Sheldrick, 1996)	*k* = −11→11
*T*_min_ = 0.690, *T*_max_ = 0.849	*l* = −11→11
5184 measured reflections	

### Refinement

**Table:**

Refinement on *F*^2^	Secondary atom site location: difference Fourier map
Least-squares matrix: full	Hydrogen site location: inferred from neighbouring sites
*R*[*F*^2^ > 2σ(*F*^2^)] = 0.038	H-atom parameters constrained
*wR*(*F*^2^) = 0.113	*w* = 1/[σ^2^(*F*_o_^2^) + (0.0645*P*)^2^ + 0.6988*P*] where *P* = (*F*_o_^2^ + 2*F*_c_^2^)/3
*S* = 1.05	(Δ/σ)_max_ < 0.001
2576 reflections	Δρ_max_ = 0.51 e Å^−^^3^
151 parameters	Δρ_min_ = −0.67 e Å^−^^3^
Primary atom site location: structure-invariant direct methods	Extinction correction: none

### Special details

**Table:**

Geometry. All e.s.d.'s (except the e.s.d. in the dihedral angle between two l.s. planes) are estimated using the full covariance matrix. The cell e.s.d.'s are taken into account individually in the estimation of e.s.d.'s in distances, angles and torsion angles; correlations between e.s.d.'s in cell parameters are only used when they are defined by crystal symmetry. An approximate (isotropic) treatment of cell e.s.d.'s is used for estimating e.s.d.'s involving l.s. planes.
Refinement. Refinement of *F*^2^ against ALL reflections. The weighted *R*-factor *wR* and goodness of fit *S* are based on *F*^2^, conventional *R*-factors *R* are based on *F*, with *F* set to zero for negative *F*^2^. The threshold expression of *F*^2^ > σ(*F*^2^) is used only for calculating *R*-factors(gt) *etc*. and is not relevant to the choice of reflections for refinement. *R*-factors based on *F*^2^ are statistically about twice as large as those based on *F*, and *R*- factors based on ALL data will be even larger.

### Fractional atomic coordinates and isotropic or equivalent isotropic displacement parameters (Å^2^)

**Table:**

	*x*	*y*	*z*	*U*_iso_*/*U*_eq_	
Zn1	0.5000	0.0000	0.0000	0.02942 (16)	
Cl1	0.18939 (10)	0.17479 (10)	0.43496 (10)	0.0530 (2)	
O1	0.3835 (2)	0.1347 (2)	−0.0573 (2)	0.0385 (5)	
H1W	0.3888	0.2257	0.0269	0.058*	
H2W	0.3035	0.0961	−0.1235	0.058*	
O2	0.6192 (2)	0.2238 (2)	0.1987 (2)	0.0445 (5)	
H3W	0.6176	0.2521	0.2964	0.067*	
H4W	0.6833	0.2949	0.1930	0.067*	
O3	0.3580 (2)	−0.0281 (3)	0.1463 (3)	0.0449 (5)	
H5W	0.3405	0.0629	0.2078	0.067*	
H6W	0.3658	−0.0839	0.1907	0.067*	
O4	0.1961 (2)	0.5028 (3)	0.7778 (3)	0.0453 (5)	
H7W	0.2082	0.4201	0.6931	0.068*	
H8W	0.1053	0.4939	0.7784	0.068*	
O5	0.1487 (3)	0.0521 (4)	0.7002 (4)	0.0734 (8)	
H9W	0.1699	0.0950	0.6431	0.110*	
H10W	0.0600	−0.0024	0.6715	0.110*	
N1	0.3345 (3)	0.7407 (3)	0.2554 (3)	0.0330 (5)	
N2	0.3362 (3)	0.6543 (3)	0.4602 (3)	0.0333 (5)	
N3	0.3418 (3)	0.4524 (3)	0.1728 (3)	0.0321 (5)	
N4	0.1152 (2)	0.5427 (3)	0.2441 (3)	0.0340 (5)	
C1	0.3865 (3)	0.7935 (3)	0.4289 (3)	0.0356 (6)	
H1A	0.3492	0.8886	0.5010	0.043*	
H1B	0.4942	0.8302	0.4552	0.043*	
C2	0.3944 (3)	0.5116 (3)	0.3488 (3)	0.0342 (6)	
H2A	0.3628	0.4191	0.3677	0.041*	
H2B	0.5022	0.5466	0.3743	0.041*	
C3	0.1782 (3)	0.4021 (3)	0.1376 (3)	0.0381 (6)	
H3A	0.1440	0.3088	0.1546	0.046*	
H3B	0.1422	0.3632	0.0223	0.046*	
C4	0.1706 (3)	0.6838 (4)	0.2176 (4)	0.0375 (6)	
H4A	0.1348	0.6480	0.1032	0.045*	
H4B	0.1311	0.7778	0.2876	0.045*	
C5	0.3925 (3)	0.5955 (4)	0.1482 (3)	0.0353 (6)	
H5A	0.3595	0.5592	0.0331	0.042*	
H5B	0.5003	0.6306	0.1729	0.042*	
C6	0.1728 (3)	0.5995 (4)	0.4186 (3)	0.0369 (6)	
H6A	0.1333	0.6925	0.4910	0.044*	
H6B	0.1386	0.5076	0.4377	0.044*	

### Atomic displacement parameters (Å^2^)

**Table:**

	*U*^11^	*U*^22^	*U*^33^	*U*^12^	*U*^13^	*U*^23^
Zn1	0.0395 (3)	0.0233 (2)	0.0250 (2)	0.00991 (17)	0.00711 (17)	0.01148 (18)
Cl1	0.0591 (5)	0.0399 (4)	0.0534 (5)	0.0135 (3)	0.0246 (4)	0.0173 (4)
O1	0.0604 (11)	0.0278 (9)	0.0329 (10)	0.0192 (8)	−0.0009 (8)	0.0105 (8)
O2	0.0710 (13)	0.0280 (10)	0.0220 (9)	−0.0076 (9)	0.0009 (9)	0.0081 (8)
O3	0.0675 (14)	0.0422 (11)	0.0548 (12)	0.0327 (10)	0.0401 (11)	0.0367 (10)
O4	0.0398 (11)	0.0386 (11)	0.0417 (11)	0.0003 (9)	0.0055 (9)	0.0130 (9)
O5	0.0604 (16)	0.086 (2)	0.0720 (18)	0.0027 (14)	−0.0156 (13)	0.0485 (16)
N1	0.0423 (12)	0.0298 (11)	0.0371 (12)	0.0160 (10)	0.0161 (10)	0.0214 (10)
N2	0.0426 (12)	0.0285 (11)	0.0242 (10)	0.0036 (9)	0.0050 (9)	0.0125 (9)
N3	0.0407 (12)	0.0255 (11)	0.0275 (11)	0.0144 (9)	0.0046 (9)	0.0102 (9)
N4	0.0345 (11)	0.0289 (11)	0.0335 (11)	0.0088 (9)	0.0063 (9)	0.0124 (9)
C1	0.0433 (15)	0.0221 (12)	0.0348 (14)	0.0048 (10)	0.0100 (11)	0.0110 (11)
C2	0.0432 (15)	0.0284 (13)	0.0326 (13)	0.0090 (11)	0.0007 (11)	0.0178 (11)
C3	0.0417 (15)	0.0264 (13)	0.0327 (14)	0.0086 (11)	−0.0005 (11)	0.0066 (11)
C4	0.0443 (15)	0.0376 (15)	0.0404 (15)	0.0219 (12)	0.0136 (12)	0.0226 (12)
C5	0.0452 (15)	0.0399 (15)	0.0314 (13)	0.0217 (12)	0.0167 (11)	0.0214 (12)
C6	0.0427 (15)	0.0337 (14)	0.0326 (14)	0.0055 (11)	0.0128 (11)	0.0167 (11)

### Geometric parameters (Å, °)

**Table:**

Zn1—O2^i^	2.0269 (18)	N2—C2	1.476 (3)
Zn1—O2	2.0269 (18)	N2—C1	1.481 (3)
Zn1—O1	2.0507 (17)	N3—C3	1.472 (4)
Zn1—O1^i^	2.0507 (17)	N3—C5	1.476 (3)
Zn1—O3^i^	2.0595 (18)	N3—C2	1.477 (3)
Zn1—O3	2.0595 (18)	N4—C4	1.477 (4)
O1—H1W	0.8223	N4—C3	1.478 (3)
O1—H2W	0.8281	N4—C6	1.479 (4)
O2—H3W	0.8279	C1—H1A	0.9700
O2—H4W	0.8360	C1—H1B	0.9700
O3—H5W	0.8221	C2—H2A	0.9700
O3—H6W	0.8267	C2—H2B	0.9700
O4—H7W	0.8326	C3—H3A	0.9700
O4—H8W	0.8374	C3—H3B	0.9700
O5—H9W	0.8338	C4—H4A	0.9700
O5—H10W	0.8316	C4—H4B	0.9700
N1—C1	1.470 (4)	C5—H5A	0.9700
N1—C4	1.476 (4)	C5—H5B	0.9700
N1—C5	1.479 (3)	C6—H6A	0.9700
N2—C6	1.471 (4)	C6—H6B	0.9700
			
O2^i^—Zn1—O2	180.00 (11)	C3—N4—C6	107.8 (2)
O2^i^—Zn1—O1	92.66 (8)	N1—C1—N2	111.7 (2)
O2—Zn1—O1	87.34 (8)	N1—C1—H1A	109.3
O2^i^—Zn1—O1^i^	87.34 (8)	N2—C1—H1A	109.3
O2—Zn1—O1^i^	92.66 (8)	N1—C1—H1B	109.3
O1—Zn1—O1^i^	180.00 (12)	N2—C1—H1B	109.3
O2^i^—Zn1—O3^i^	89.59 (9)	H1A—C1—H1B	107.9
O2—Zn1—O3^i^	90.41 (9)	N2—C2—N3	111.6 (2)
O1—Zn1—O3^i^	86.57 (8)	N2—C2—H2A	109.3
O1^i^—Zn1—O3^i^	93.43 (8)	N3—C2—H2A	109.3
O2^i^—Zn1—O3	90.41 (9)	N2—C2—H2B	109.3
O2—Zn1—O3	89.59 (9)	N3—C2—H2B	109.3
O1—Zn1—O3	93.43 (8)	H2A—C2—H2B	108.0
O1^i^—Zn1—O3	86.57 (8)	N3—C3—N4	112.2 (2)
O3^i^—Zn1—O3	180.00 (19)	N3—C3—H3A	109.2
Zn1—O1—H1W	109.5	N4—C3—H3A	109.2
Zn1—O1—H2W	126.6	N3—C3—H3B	109.2
H1W—O1—H2W	113.2	N4—C3—H3B	109.2
Zn1—O2—H3W	124.5	H3A—C3—H3B	107.9
Zn1—O2—H4W	124.2	N1—C4—N4	112.2 (2)
H3W—O2—H4W	111.0	N1—C4—H4A	109.2
Zn1—O3—H5W	109.6	N4—C4—H4A	109.2
Zn1—O3—H6W	123.5	N1—C4—H4B	109.2
H5W—O3—H6W	113.5	N4—C4—H4B	109.2
H7W—O4—H8W	110.1	H4A—C4—H4B	107.9
H9W—O5—H10W	111.5	N3—C5—N1	111.7 (2)
C1—N1—C4	108.4 (2)	N3—C5—H5A	109.3
C1—N1—C5	108.2 (2)	N1—C5—H5A	109.3
C4—N1—C5	108.3 (2)	N3—C5—H5B	109.3
C6—N2—C2	108.5 (2)	N1—C5—H5B	109.3
C6—N2—C1	108.5 (2)	H5A—C5—H5B	108.0
C2—N2—C1	108.0 (2)	N2—C6—N4	112.0 (2)
C3—N3—C5	108.7 (2)	N2—C6—H6A	109.2
C3—N3—C2	108.4 (2)	N4—C6—H6A	109.2
C5—N3—C2	107.9 (2)	N2—C6—H6B	109.2
C4—N4—C3	108.0 (2)	N4—C6—H6B	109.2
C4—N4—C6	108.1 (2)	H6A—C6—H6B	107.9
			
C4—N1—C1—N2	58.4 (3)	C1—N1—C4—N4	−58.6 (3)
C5—N1—C1—N2	−58.8 (3)	C5—N1—C4—N4	58.6 (3)
C6—N2—C1—N1	−58.6 (3)	C3—N4—C4—N1	−58.3 (3)
C2—N2—C1—N1	58.9 (3)	C6—N4—C4—N1	58.1 (3)
C6—N2—C2—N3	58.3 (3)	C3—N3—C5—N1	58.1 (3)
C1—N2—C2—N3	−59.1 (3)	C2—N3—C5—N1	−59.2 (3)
C3—N3—C2—N2	−58.2 (3)	C1—N1—C5—N3	59.2 (3)
C5—N3—C2—N2	59.4 (3)	C4—N1—C5—N3	−58.1 (3)
C5—N3—C3—N4	−58.3 (3)	C2—N2—C6—N4	−58.8 (3)
C2—N3—C3—N4	58.7 (3)	C1—N2—C6—N4	58.4 (3)
C4—N4—C3—N3	58.0 (3)	C4—N4—C6—N2	−58.0 (3)
C6—N4—C3—N3	−58.6 (3)	C3—N4—C6—N2	58.5 (3)

Symmetry codes: (i) −*x*+1, −*y*, −*z*.

### Hydrogen-bond geometry (Å, °)

**Table:**

*D*—H···*A*	*D*—H	H···*A*	*D*···*A*	*D*—H···*A*
O1—H1W···N3	0.82	2.05	2.827 (3)	158
O1—H2W···O5^ii^	0.83	1.94	2.743 (3)	162
O2—H3W···N2^iii^	0.83	1.99	2.804 (3)	167
O2—H4W···O4^iii^	0.84	1.90	2.711 (3)	165
O3—H5W···Cl1	0.82	2.55	3.197 (2)	137
O3—H6W···N1^iv^	0.83	2.01	2.813 (3)	165
O4—H7W···Cl1	0.83	2.36	3.175 (2)	168
O4—H8W···N4^v^	0.84	2.00	2.835 (3)	174
O5—H9W···Cl1	0.83	2.43	3.255 (3)	168
O5—H10W···Cl1^vi^	0.83	2.38	3.213 (3)	175

Symmetry codes: (ii) *x*, *y*, *z*−1; (iii) −*x*+1, −*y*+1, −*z*+1; (iv) *x*, *y*−1, *z*; (v) −*x*, −*y*+1, −*z*+1; (vi) −*x*, −*y*, −*z*+1.
